# Long-Term Effects of Alemtuzumab on CD4+ Lymphocytes in Multiple Sclerosis Patients: A 72-Month Follow-Up

**DOI:** 10.3389/fimmu.2022.818325

**Published:** 2022-02-28

**Authors:** Simona Rolla, Stefania Federica De Mercanti, Valentina Bardina, Alessandro Maglione, Daniela Taverna, Francesco Novelli, Eleonora Cocco, Anton Vladic, Mario Habek, Ivan Adamec, Pietro Osvaldo Luigi Annovazzi, Dana Horakova, Marinella Clerico

**Affiliations:** ^1^ Department of Clinical and Biological Sciences, University of Torino, Torino, Italy; ^2^ Laboratory of Microbiology and Virology, Amedeo di Savoia Hospital, Torino, Italy; ^3^ Department of Molecular Biotechnology and Health Sciences, University of Torino, Torino, Italy; ^4^ Department of Medical Science and Public Health, University of Cagliari and Multiple Sclerosis Center, Cagliari, Italy; ^5^ Department of Neurology, Clinical Hospital Sveti Duh Zagreb and Medical Faculty, University J.J Strossmayer Osijek, Prague, Croatia; ^6^ Referral Center for Autonomic Nervous System, University Hospital Center Zagreb, Zagreb, Croatia; ^7^ School of Medicine, University of Zagreb, Zagreb, Croatia; ^8^ Multiple Sclerosis Centre, Gallarate Hospital, ASST Valle Olona, Gallarate, Italy; ^9^ Department of Neurology and Center of Clinical Neuroscience, First Faculty of Medicine, Charles University and General University Hospital, Prague, Czechia

**Keywords:** multiple sclerosis, alemtuzumab, immune reconstitution, Treg cells, MBP (myelin basic protein)

## Abstract

**Introduction:**

Alemtuzumab is highly effective in the treatment of patients with relapsing multiple sclerosis (PwRMS) and selectively targets the CD52 antigen, with a consequent profound lymphopenia, particularly of CD4+ T lymphocytes. However, the immunological basis of its long-term efficacy has not been clearly elucidated.

**Methods:**

We followed up 29 alemtuzumab-treated RMS patients over a period of 72 months and studied the immunological reconstitution of their CD4+ T cell subsets by means of phenotypic and functional analysis and through mRNA-related molecule expression, comparing them to healthy subject (HS) values (rate 2:1).

**Results:**

In patients receiving only two-course alemtuzumab, the percentage of CD4+ lymphocytes decreased and returned to basal levels only at month 48. Immune reconstitution of the CD4+ subsets was characterized by a significant increase (p < 0.001) in Treg cell percentage at month 24, when compared to baseline, and was accompanied by restoration of the Treg suppressor function that increased within a range from 2- to 6.5-fold compared to baseline and that persisted through to the end of the follow-up. Furthermore, a significant decrease in self-reactive myelin basic protein-specific Th17 (p < 0.0001) and Th1 (p < 0.05) cells reaching HS values was observed starting from month 12. There was a change in mRNA of cytokines, chemokines, and transcriptional factors related to Th17, Th1, and Treg cell subset changes, consequently suggesting a shift toward immunoregulation and a reduction of T cell recruitment to the central nervous system.

**Conclusions:**

These data provide further insight into the mechanism that could contribute to the long-term 6-year persistence of the clinical effect of alemtuzumab on RMS disease activity.

## Introduction

Alemtuzumab, a monoclonal antibody that targets the CD52 antigen, is the first immune reconstitution therapy in Europe and the USA to be approved for patients with relapsing multiple sclerosis (PwRMS). Alemtuzumab administration determines a rapid and marked reduction in peripheral T and B lymphocytes, which express CD52 molecules at high levels on their membrane, due to antibody-dependent cell-mediated cytotoxicity, complement-dependent cytolysis, and induction of apoptosis (1) with a subsequent beneficial reconstitution of the immune system (2). The lack of CD52 expression on bone marrow-derived hematopoietic cells enables immune reconstitution, which is obtained over several months (3, 4), and return of immune competency (5). Specific immune repopulation patterns appear to be responsible for the long-term efficacy of alemtuzumab that persists even years after the last course of therapy: B lymphocytes recover first, followed by CD8+ and CD4+ T lymphocytes (2–6). As immune reconstitution proceeds, Tregs represent the majority of the T lymphocyte population and thus are believed to be one of the reasons for long-term alemtuzumab effectiveness (6–8).

We had previously organized a multicenter 24-month study (7) to analyze the changes in Th subsets, Treg proportion and function, and mRNA levels of cytokines and other immunologically related molecules in 29 patients from phase III trials CARE-MS I (3) and CARE-MS II (4). The data showed a different T cell repopulation among the CD4+ T cells: while the percentage of Th1 and Th17 cells did not have any relevant change, a significant increase in Treg cell percentage with restored suppressive function was observed at 24 months post treatment. Moreover, mRNA levels of pro-inflammatory and anti-inflammatory cytokines were downregulated and upregulated respectively following treatment, which may also favor the drug’s long-term efficacy in RMS (7, 9). In this paper, we herewith report the now complete long-term follow-up of 72 months focusing on the study of the CD4+ immune cell reconstitution in those 24 patients who had received the two classical alemtuzumab administrations at months 0 and 12, and studying the CD4+ immune cell reconstitution so as to compare it to the healthy subjects (HS).

## Materials and Methods

### Patients and Clinical Study Design

Twenty-nine PwRMS participating in CARE-MS I (3) and II (4) trials in 6 European MS centers were enrolled and evaluated at baseline and for 72 months after alemtuzumab treatment. Inclusion and exclusion criteria were described in the original articles (3, 4). Patients were treated with 12 mg/d IV alemtuzumab in 2 annual courses (5 administrations at month 0 and 3 administrations at month 12). Patients’ demographic and clinical characteristics are reported in **Table 1**. Neurologic assessments, performed by blinded investigators, were done at baseline and repeated every month or in case of relapses. Clinical data were collected in clinical research forms (CRF) and sent to the coordinating center located at the University of Torino. Blood samples were taken at baseline (before the first alemtuzumab course) and at months 6, 12 (before the second alemtuzumab course), 18, 24, 36, 48, 60, and 72. Fresh blood was collected in heparin-treated vacutainers and immediately sent to the coordinating center located at the University of Torino for immunologic testing. All samples were received and processed within 48 h from the blood withdrawal. Twelve sex- and age-matched healthy subjects were also enrolled from every center (rate cases/controls: 2:1).

**Table 1:** Demographic and clinical data.

Demographic data		
	PwRMS	HS
No. of subject	29	12
Sex (% of female)	58%	62%
Age at baseline	34.0 ± 8.7	31.0 ± 5.7
Previous treatment	IFNβ (27). GA (1). none (1)	–
Disease duration (years)	5.0 ± 3.4	–
EDSS at baseline	2.0 (1.5–3.5)	–
EDSS at month 72	1.7 (1.5–3.5)	–
Number of patients who experienced relapses	13	–
Numbers of relapses in the 6-year follow up	1.6 ± 0.9	–
Number of patients who developed secondary autoimmunity	9	–

Values are percentages or mean ± SD and median and interquartile range for EDSS.

### Standard Protocol Approvals, Registrations, and Patient Consents

The institutional review board of the participating centers approved the study, and all subjects gave written informed consent (protocol number Bio2009001).

### Flow Cytometry (Fluorescence-Activated Cell Sorting)

We used the same methodology as the one employed in our previous study (7). Peripheral blood mononuclear cells (PBMC) were isolated by density gradient centrifugation from heparinized venous blood. PBMC were stained for Treg cells with anti-CD4, anti-CD25, anti-CD127, anti-CD45RO, and anti-CD45RA monoclonal antibodies (mAb) (BioLegend, San Diego, CA) on the cell surface. For detection of the transcriptional factor FoxP3, cells were fixed with fixation and permeabilization buffers (eBioscience, San Diego, CA). PBMC were cultured in Iscove’s modified Dulbecco’s medium (BioWhittaker, Walkersville, MD) supplemented with 10% fetal bovine serum (Invitrogen, Carlsbad, CA) and stimulated for 5 h with phorbol 12-myristate 13-acetate PMA (50 ng/ml) and ionomycin (500 ng/ml) in the presence of brefeldin A (10 mg/ml, Sigma-Aldrich, St. Louis, MO). Cells were first stained for the surface antigen CD4 (BioLegend) and then fixed with 4% paraformaldehyde, permeabilized with 0.5% saponin, followed by intracellular staining with anti-IL-17 and anti-IFN-γ mAbs (BioLegend) (10, 11). Stained PBMC were acquired on a FACSCalibur (BD Biosciences, San Jose, CA) and analyzed with FlowJo software (Ashland, OR). For detection of Treg cells, stained PBMC were first gated on CD4 and CD25. CD4+CD25^high^ T cells were analyzed for co-expression of FOXP3 and CD127low identifying CD4+CD25^high^CD127^low^FOXP3+Tregs (**Supplementary Figure 1A**). Tregs were then analyzed for expression of CD45RA and CD45R0. For detection of Th17 and Th1 cells, stained PBMC were first gated on CD4 and then analyzed for IL-17 or IFN-γ production (**Supplementary Figure 1B**). Absolute values of Treg, Th17, and Th1 cells were calculated normalizing the percentage of CD25^high^CD127^low^FOXP3+, IL-17-producing cells, and IFN-γ-producing cells on the CD4 T cell count obtained from complete blood count with formula and expressed as cells/μL of blood.

### Cytokine mRNA Analysis

We used the same methodology as the one employed in our previous study (7). Aliquots (0.5 ml each) of blood were mixed with 1.3 ml RNAlater (Ambion, Life Technologies, Carlsbad, CA) immediately after arrival at the coordinating canter and stored at 280°C. To determine mRNA levels, samples were treated as previously described (12): RNA was extracted with the RiboPure Blood Kit (Ambion, Foster City, CA, USA) and cDNA obtained with the High-Capacity cDNA Reverse Transcription Kit (Applied Biosystems, Life Technologies, Monza, Italy) (12).

We assessed the mRNA levels of the following 26 immunologically relevant molecules whose function in MS has been documented by using TaqMan low-density arrays (Applied Biosystems 7900HT Real-Time PCR System) (12): molecules with pro-inflammatory function including IL-1b, IL-2, IL-6, IL-12, IL-17A, IL-17F, IL-21, IL-22, IL-23, IL-26, IFN-γ, T-box expressed in T cells (Tbet), retinoid-related orphan receptor g (RORC), tumor necrosis factor-a (TNF-α), C–C chemokine receptor type 3 (CCR3), CCR4, CCR5, CCR6, C–X–C chemokine receptor type 3 (CXCR3), C–X–C motif ligand 10 (CXCL10), C–C motif ligand 20 (CCL20), and very late antigen 4 (VLA4), and molecules with anti-inflammatory function, including IL-10, IL-27, transforming growth factor–b (TGF-β), and forkhead box P3 (FoxP3). Glyceraldehyde-3 phosphate dehydrogenase served as the housekeeping gene for normalization. The relative expression of each gene was calculated using the comparative threshold cycle method as directed by the manufacturer (User Bulletin No. 2, Applied Biosystems, Foster City, CA, USA) and expressed in arbitrary units as previously described in detail (7).

### Enzyme-Linked Immunospot Assay

Antigen-specific IFN-γ and IL-17-producing cells and antigen-specific suppressor activity of Treg cells were assessed by enzyme-linked immunospot (ELISPOT) (eBioscience) at months 0, 12, 24, 36, 48, 60, and 72. We used the same methodology as the one employed in our previous study (7). To remove Treg cells from PBMC (PBMC^CD25-^), the CD25^+^ fraction was depleted using immunomagnetic beads (CD4^+^ CD25^+^ Regulatory T Cell Isolation Kit, Miltenyi Biotec, Bergisch Gladbach, Germany). The purity of the depleted fraction was immediately analyzed by fluorescence-activated cell sorting (FACS) staining and ranged from 92 to 95%. 1 × 10^5^ total PBMC or PBMC^CD25-^ was seeded in 96-well ELISPOT assay plates (Millipore, Darmstadt, Germany) in triplicate and incubated for 48 h at 37°C either with myelin basic protein (MBP, 40 mg/ml, Sigma-Aldrich), with the purified protein derivative of tuberculin (PPD, 40 mg/ml, Sigma-Aldrich) as negative control, or with anti-CD3 and anti-CD28 mAbs (10 and 1 μg/ml, respectively) as an internal test control. IFN-γ-specific or IL-17-specific spots were counted by computer-assisted image analysis (Transtec 1300 ELISpot Reader; AMI Bioline, Buttigliera Alta, Italy). The number of IFN-γ or IL-17 spots produced spontaneously was subtracted from the number of spots produced by antigen-stimulated cells to obtain the number of IFN-γ and IL-17 antigen-specific spots in the PBMC or in the PBMC^CD25-^, as previously described (7). Median values for the triplicates, adjusted as the number of IL-17- and IFN-γ-producing cells/10^6^ PBMC, were used.

### Statistical Analysis

Statistical analysis was performed using GraphPad Prism 8.0 (La Jolla, CA) software. For the longitudinal follow-up, statistical significance was calculated concerning baseline and to HS by using one-way analysis of variance for repeated measures followed by the Bonferroni multiple-comparison post-test. The Pearson t-test was used to analyze the differences between groups. p values < 0.05 were considered statistically significant.

## Results

### Clinical Characteristics of the Cohort

This study represents the continuation of the one previously published (7). Thirteen patients experienced a total of 16 clinical relapses (at months 1, 9, 10, 12, 20, 25, 28, 29, 30, 36, 41, 70) which brought 5 patients to receive additional courses of the drug. The median of Expanded Disease Status Scale (EDSS) score did not change significantly during the 72-month follow-up (EDSS median score from 2 at baseline to 1, 7 at month 72). Secondary autoimmune thyroiditis occurred in 9 patients at months 24, 30, 36, 42, and 59.

Of the 29 patients recruited in this study, only patients who had received the “classical” two courses of alemtuzumab were analyzed in this new study (24 patients). Two patients, described in a different paper (13), were excluded from the analysis due to their atypical CD4+ T population behavior after alemtuzumab administration: they showed persistent disease activity despite repeated alemtuzumab treatment and, while lymphocyte count decreased and fluctuated according to alemtuzumab administration, their CD4+ cell percentage was not affected or was barely affected and was slightly below the lowest normal limit prior to alemtuzumab (13). The other 3 patients had been excluded as they had received a third course of alemtuzumab at month 30 or 36 due to a relapse.

### Pro-Inflammatory Th17 and Th1 Cells and Their Related Molecules Decreased Persistently Throughout the 72 Months

**Figure 1:**
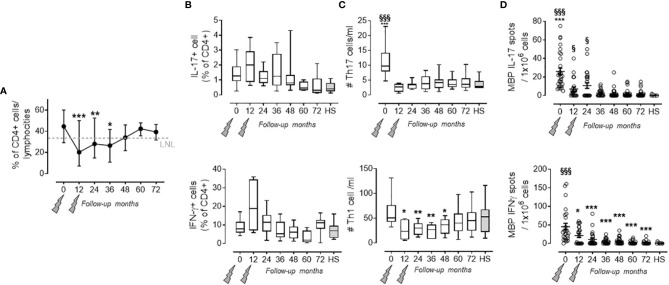
Pro-inflammatory Th17 and Th1 cells in the PB of PwRMS treated with alemtuzumab and in HS. **(A)** % of CD4 lymphocytes on total lymphocytes. **(B)** Th17 and Th1 cell (identified as IL-17-producing CD4+ T cells and IFN-γ-producing CD4+ T cells, respectively) percentage among the CD4+ fraction. **(C)** Absolute numbers of Th17 and Th1 cells circulating in the PB. **(D)** IL-17 and IFN-γ MBP-specific spots in the PBMC. Months after alemtuzumab administration and HS are indicated in the X-axis. Whiskers represent minimum to maximum values. Statistical significance (one-way ANOVA) was calculated with respect to month 0 (*p < 0.05; **p < 0.001; ***p < 0.0001) and to HS (^§^p < 0.05; ^§§§^p < 0.0001, gray bars and dots). The arrows indicate infusion of alemtuzumab.

As previously observed, the percentage of CD4+ T cells in the PBMC rapidly decreased after the first administration (7) course and returned to the lowest normal limit (LNL) only at month 48 (13), and this was maintained over months 60 (40.78 ± 2.97%) and 72 (39.37 ± 2.40%) where the LNL was 34 (**Figure 1A**). Within the CD4+ cell fraction, we did not observe any significant change in the percentage of pro-inflammatory Th17 and Th1 cells (defined by the production of IL-17 and IFN-γ, respectively, in the CD4+ T cell fraction) at any time point of the follow-up, when compared to HS (**Figure 1B**). However, the absolute number of circulating Th17 had significantly decreased after alemtuzumab (i.e., month 0: 1,100 ± 610; month 24: 380 ± 300; month 72: 410 ± 270 cells/ml) compared to the number obtained in HS (270 ± 210 cells/ml; **Figure 1C** upper panel). The absolute number of circulating Th1 had significantly decreased after alemtuzumab until month 48, compared to baseline (6,341 ± 3,182 at month 0 vs. 3,422 ± 1,581 cells/ml at month 48), but all the values assessed during the follow-up were not different from those of HS (5,292 ± 3,378 cells/ml; **Figure 1C** lower panel). To better evaluate the immune response involved in RMS, we also evaluated the antigen-specific response directed against MBP. One should note that MBP-specific IL-17-producing cells and IFN-γ-producing cells significantly decreased after alemtuzumab administration and were comparable (not statistically different) to HS starting from months 36 (2.85 ± 3.79 vs. 0.27 ± 0.64 IL-17 spots) and 12 (21.50 ± 23.47 vs. 1.1 ± 2.03 IFN-γ spots), respectively (**Figure 1D**).

Accordingly, mRNA levels of pro-inflammatory cytokines produced by Th17 cells, IL-17A (14), IL-17F (14), IL-21 (15), IL-22 (11), IL-26 (16), and by Th1 cells, IFN-γ (17), and their related transcriptional factors, RORC (18) and Tbet (19), significantly declined to the values of HS at each time point of the follow-up when compared to baseline (**Table 2**). A similar behavior was observed for their chemokines and chemokine receptors, CCL20 (20) and CXCL10 (21), CCR6 (22), CCR5, and CXCR3 (23), involved in T cell recruitment into the CNS (**Table 2**). Alemtuzumab administration also reduced mRNA levels of cytokines known to guide Th17 [IL-1β (24), IL-6 (25), IL-23 (26)], and Th1 [IL-12 (27)] differentiation, but their levels remained higher compared to HS, suggesting that a pro-inflammatory environment could persist in these 24 patients.

**Table 2:** mRNA levels of pro- and anti-inflammatory molecules evaluated at months 0, 12, 24, 36, 48, 60, and 72 after alemtuzumab administration and in HS.

	0	12	24	36	48	60	72	HS
*IL-17A*	50.50 ± 2.50 ^§§§^	0.05 ± 0.01 ***	0.01 ± 0.01 ***	0.02 ± 0.01 ***	0.02 ± 0.01 ***	0.01 ± 0.01 ***	0.01 ± 0.01 ***	0.01 ± 0.01
*IL-17F*	25.60 ± 10.7 ^§§§^	0.03 ± 0.01 ***	0.03 ± 0.01 ***	nd ***	nd ***	nd ***	Nd ***	0.36 ± 0.80
*IL-21*	0.02 ± 0.02 ^§§§^	nd ***	nd ***	nd ***	nd ***	nd ***	Nd ***	0.48 ± 0.19
*IL-22*	15.58 ± 3.10 ^§§§^	0.05 ± 0.01 ***	0.04 ± 0.01 ***	0.05 ± 0.01 ***	0.05 ± 0.01 ***	nd ***	Nd ***	0.96 ± 0.58
*IL-23*	374.00 ± 6.70 ^§§§^	76.00 ± 2.00 *** ^§§§^	65.42 ± 3.80 *** ^§§§^	67.34 ± 2.10 *** ^§§§^	69.07 ± 19.05 *** ^§§§^	60.81 ± 2.70 *** ^§§§^	59.31 ± 5.30 *** ^§§§^	0.58 ± 0.38
*IL-26*	11.39 ± 1.87 ^§§§^	0.01 ± 0.01 ***	0.01 ± 0.01 ***	nd ***	nd ***	nd ***	Nd ***	0.36 ± 0.11
*IL-1β*	373.60 ± 9.30 ^§§§^	88.70 ± 2.20 *** ^§§§^	88.70 ± 2.20 *** ^§§§^	64.20 ± 2.60 *** ^§§§^	69.80 ± 2.70 *** ^§§§^	62.26 ± 3.30 *** ^§§§^	63.15 ± 3.17 *** ^§§§^	1.10 ± 0.22
*IL-6*	503.80 ± 9.30 ^§§§^	47.20 ± 2.30 *** ^§§^	56.100 ± 1.3 *** ^§§^	55.13 ± 1.50 *** ^§§^	52.53 ± 1.80 *** ^§§^	47.32 ± 2.20 *** ^§§^	51.73 ± 2.10 *** ^§§^	0.02 ± 0.01
*TNF-α*	830.60 ± 18.70 ^§§§^	96.07 ± 2.40 *** ^§§^	72.90 ± 2.70 *** ^§§^	74.44 ± 2.50 *** ^§§^	72.10 ± 3.40 *** ^§§^	76.64 ± 5.10 *** ^§§^	69.54 ± 3.7 *** ^§§^	0.21 ± 0.03
*RORC*	11.30 ± 0.60 ^§§§^	0.01 ± 0.01 ***	0.01 ± 0.01 ***	nd ***	nd ***	nd ***	Nd ***	0.01 ± 0.01
*CCR6*	473.70 ± 24.80 ^§§§^	80.90 ± 1.80 *** ^§§^	82.65 ± 5.70 *** ^§§^	77.90 ± 5.30 *** ^§§^	71.90 ± 4.20 *** ^§§^	16.90 ± 3.50 *** ^§§^	17.23 ± 3.80 *** ^§§^	0.24 ± 0.10
*CCL20*	23.50 ± 2.05 ^§§§^	0.80 ± 0.07 ***	0.01 ± 0.01 ***	0.10 ± 0.01 ***	nd ***	nd ***	Nd ***	0.10 ± 0.01
*INF-γ*	217.10 ± 6.10 ^§§§^	51.50 ± 2.20 ***	35.80 ± 1.40 ***	38.30 ± 3.80 ***	36.10 ± 1.20 ***	35.50 ± 3.20 ***	32.80 ± 1.40 ***	49.30 ± 5.10
*IL-12*	436.40 ± 11.41 ^§§§^	93.20 ± 2.20 *** ^§§§^	69.40 ± 2.40 *** ^§§§^	66.20 ± 2.50 *** ^§§§^	64.70 ± 3.10 *** ^§§§^	58.12 ± 4.50 *** ^§§§^	61.70 ± 3.91 *** ^§§§^	1.10 ± 0.06
*Tbet*	764.30 ± 17.00 ^§§§^	40.40 ± 1.60 ***	24.58 ± 2.30 ***	26.21 ± 2.00 ***	25.47 ± 2.10 ***	29.50 ± 3.90 ***	20.70 ± 1.60 ***	1.10 ± 0.11
*CCR5*	77.50 ± 5.10 ^§§§^	19.41 ± 0.90 ***	11.82 ± 1.40 ***	12.92 ± 1.30 ***	13.80 ± 1.11 ***	11.42 ± 1.91 ***	10.30 ± 1.80 ***	11.30 ± 0.60
*CXCR-3*	150.01 ± 4.70 ^§§§^	53.30 ± 1.70 ***	54.10 ± 5.10 ***	46.6 ± 1.8 ***	42.61 ± 2.10 ***	36.70 ± 6.11 ***	26.11 ± 6.20 ***	0.30 ± 0.02
*CXCL-10*	72.20 ± 3.11 ^§§§^	21.50 ± 1.20 ***	22.10 ± 1.70 ***	21.50 ± 1.38 ***	21.28 ± 1.70 ***	14.80 ± 3.70 ***	14.30 ± 3.10 ***	14.20 ± 2.90
*FoxP3*	13.50 ± 0.80 ^§§§^	205.60 ± 27.11 *** ^§§§^	328.80 ± 18.10 *** ^§§§^	342.50 ± 12.10*** ^§§§^	339.40 ± 12.10 *** ^§§§^	291.30 ± 15.90*** ^§§§^	332.50 ± 10.51 *** ^§§§^	1.70 ± 0.20
*IL-10*	47.31 ± 0.70 ^§§§^	494.90 ± 14.10 *** ^§§§^	526.00 ± 16.70 *** ^§§§^	495.00 ± 18.01 *** ^§§§^	524.00 ± 23.90 *** ^§§§^	509.20 ± 13.70 *** ^§§§^	491.51 ± 21.30 *** ^§§§^	0.46 ± 0.13
*TGF-β*	46.40 ± 1.80	894.80 ± 40.10 *** ^§§§^	994.10 ± 24.10 *** ^§§§^	892.70 ± 30.31*** ^§§§^	837.80 ± 34.10*** ^§§§^	806.90 ± 36.11 *** ^§§§^	818.11 ± 28.90 *** ^§§§^	5.40 ± 0.11
*IL-27*	116.30 ± 1.80 ^§§§^	1189.01 ± 40.80 ***^§§§^	1181.00 ± 57.50 ***^§§§^	1073.0 ± 28.10 ***^§§§^	1060.00 ± 32.10 ***^§§§^	1038.0 ± 40.91 ***^§§§^	908.50 ± 79.21 ***^§§§^	4.20 ± 0.71

Results are shown as AU (see Methods) ± SEM; statistical significance was calculated by one-way ANOVA.

***p < 0.0001 compared to baseline.

^§§^p < 0.001 compared to HS.

^§§§^p < 0.0001 compared to HS.

nd, not detectable.

### Treg Cell Function Is Restored and Persists Throughout the 72-Month Follow-Up

**Figure 2:**
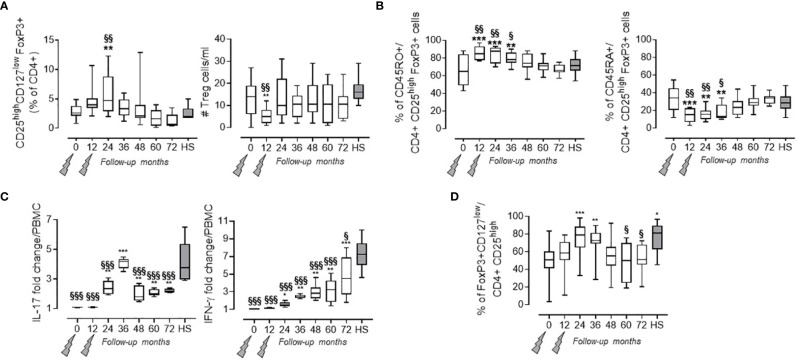
Treg cells in the PB of PwRMS treated with alemtuzumab and in HS. **(A)** Treg cells percentage among the CD4+ fraction and absolute numbers in the blood. **(B)** Percentage of Treg cells expressing CD45RO and CD45RA. Statistical significance (one-way ANOVA) was calculated with respect to month 0 (**p < 0.001) and to HS (**^§§^** p < 0.001). **(C)** % of change of MBP-specific spots in the PBMC^CD25-^ over PBMC after background subtraction of unstimulated PBMC or PBMC^CD25-^. **(D)** FoxP3 expression in Treg cells expressed as % of FoxP3 + on CD4 + CD25 + cells. Whiskers represent minimum to maximum values. Statistical significance (one-way ANOVA) was calculated with respect to month 0 (*p < 0.05; **p < 0.001; ***p < 0.0001) and to HS (^§^p < 0.05; ^§§^p < 0.001; ^§§§^p < 0.0001, gray bars and dots). Months after alemtuzumab administration and HS are indicated in the X-axis. The arrows indicate infusion of alemtuzumab.

As previously reported, a significant increase in Treg cell percentage (evaluated as CD4+ CD25^high^ CD127^low^ FoxP3+ cells) was detected at month 24 when compared to baseline (7). Afterward, the values of Treg cell percentage revert back to their basal levels which are similar to those observed in HS (i.e., month 36: 3.48 ± 1.48 in PwRMS vs. 2.55 ± 1.25% in HS; **Figure 2A**). This behavior is reflected by the Treg cell absolute count in the blood that, after an initial decrease at month 12, then reaches levels similar to HS as of month 24. Up to month 36, the majority of Treg cells exhibited a memory phenotype as indicated by the significant increase in the percentage of memory CD45RO+FoxP3+CD4+lymphocytes, accompanied by a relative contraction of the percentage of naive CD45RA+ FoxP3+CD4+ cells (**Figure 2B**). mRNA levels of Treg transcription factor FoxP3 (28), and of anti-inflammatory cytokines related to Treg subset IL-10, TGFβ (29), and IL-27 (30), had increased throughout all time points of the follow-up compared both to baseline and to HS (**Table 2**).

Treg suppressor function was assessed by measuring the MBP-specific IL-17 and IFN-γ production in the PBMC both before and after CD4+CD25+ depletion. This method allowed us to test the presence of functional Treg cells also in months with high lymphopenia, through the increase in IL-17 and IFN-γ spot production by PBMC^CD25-^. Treg suppressor function, expressed as fold change over PBMC (**Figure 2C**), was restored at month 24 (as previously observed in 7) and persisted through to the end of the follow-up, even if their functions remain significantly lower compared to HS (**Figure 2C** and **Supplementary Figure 2**). Furthermore, the increase in suppressive capacity of Treg cells was confirmed both by FoxP3 expression in Treg cells (**Figure 2D**) and by the increase of FoxP3 mRNA levels in the blood (**Table 2**).

### Correlation of Immunological Evaluation With Clinical Parameters

**Figure 3:**
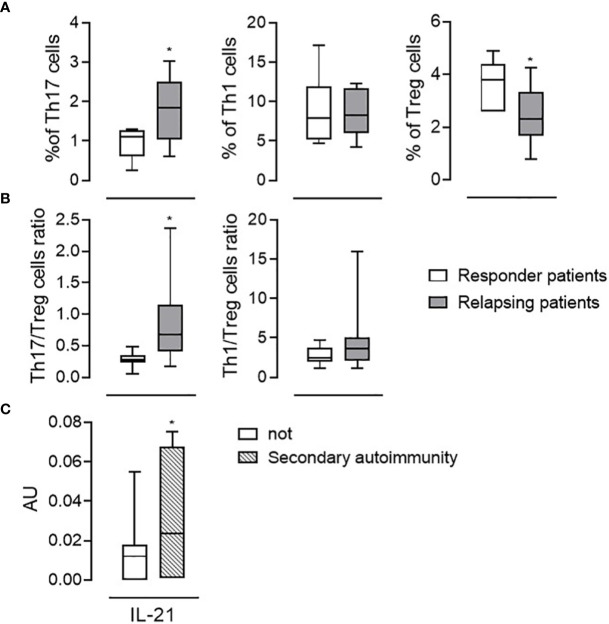
Immunological features associated with relapses or autoimmunity. Th17, Th1, and Treg cell percentage among the CD4+ fraction **(A)** and Th17/Treg Th1/Treg cell ratios **(B)** evaluated at the baseline (month 0) in responder patients (white bars, n = 16) and in patients in which relapses occurred (relapsing patients, gray bars, n = 13). **(C)** mRNA levels of IL-21 in the blood of patients who developed autoimmunity (dashed bars; n = 9) and not (white bars; n = 20). Whiskers represent minimum to maximum values. *p < 0.05, Pearson t-test.

As thirteen PwRMS had one or more relapses during the follow-up, we wondered if some of the immunological parameters we had evaluated differed in “responders” (i.e., PwRMS that did not develop any relapses) versus “relapsing PwRMS” before starting alemtuzumab. Clinical data of these subgroups are reported in **Table 3**. Interestingly, we observed a significantly higher percentage of Th17 cells (1.76 ± 0.77 vs. 0.93 ± 0.38%), but not of Th1, and a low percentage of Treg (2.40 ± 1.05 vs. 3.70 ± 0.82%) cells in “relapsing PwRMS” compared to “responders” at month 0 (**Figure 3A**). In the same way, the Th17/Treg cell ratio was highest in “relapsing PwRMS” (**Figure 3B**). The same analysis was performed on the patient who developed secondary autoimmunity; a significant increase in the mRNA levels of IL-21 was detected at baseline in the subject who developed thyroiditis compared with subjects who did not develop secondary autoimmunity (**Figure 3C**).

**Table 3:** Clinical data of responders vs. relapsing PwMS.

	Responders (n = 16)	Relapsing PwMS (n = 13)	Statistics
Age at baseline	37.13 ± 8.23	30.77 ± 8.31	p = 0.04
Sex (% of female)	56.21%	61.56%	–
Disease duration (years)	6.32 ± 3.31	5.84 ± 3.48	p = 0.67
EDSS at baseline	2.25 (1.50–3.87)	2.00 (1.50–3.50)	p = 0.76
EDSS at month 72	2.00 (1.00–3.50)	1.50 (1.50–3.75)	p = 0.92
Worst EDSS at relapse	–	3.50 (3.00–3.50)	–

Values of demographic data are percentages or mean ± SD. EDSS is indicated as median and interqualtile range. Statistcal significance was assesed by Mann-Whitney T test.

## Discussion

Alemtuzumab is a recombinant humanized immunoglobulin G1 (IgG1) monoclonal antibody directed against the CD52 antigen, a small protein of undefined function (31) expressed at a high level on the surface of T and B lymphocytes, to a lesser extent on NK cells, monocytes, macrophages, and eosinophils, while it is absent or barely expressed in neutrophils, dendritic cells (DCs), and hematopoietic stem cells (32, 33). The specific repopulation pattern of T and B cells accounts for its long-term efficacy; furthermore, growing evidence suggests a rearrangement of the T and B cell network. Here, we investigated the numbers and function of the three main subsets of CD4+ cells in PwRMS patients in a 6-year follow-up study after the classical two cycles of alemtuzumab. Our results indicate that alemtuzumab’s long-lasting therapeutic effect is associated with a reconstitution of the CD4+ T cell subsets characterized by an initial expansion of memory Treg cells and mainly by a persistent restoration of their suppressive function, accompanied by a shift in the cytokine balance from inflammation toward immune tolerance.

Th17 cells, Th1 cells, and Treg cells are amply recognized as having a pivotal role in the pathogenesis and in the course of RMS (34). Myelin-reactive Th17 and Th1 cells are probably the main effectors involved in the final pathogenetic pathway (10, 35). On the other hand, Treg cells are believed to counteract Th1 and Th17 proinflammatory effects (36) and, in the context of autoimmune diseases, they possess plasticity and instability that allow them to acquire effector-like and tissue-specific properties (37). In our previous work, we observed that the number of Th17 cells and Th1 cells in the blood and the mRNA transcript of immunological molecules related to these subsets decreased at months 6, 12, 18, and 24 after alemtuzumab administration, when compared to baseline. This peculiar effect of alemtuzumab is maintained through to month 72, in agreement with other recent reports (38, 39). Furthermore, alemtuzumab brings the number of Th17 and Th1 cells back to those present in HS. In line with these results, also the mRNA levels of cytokines produced by Th17 and Th1 cells, of cytokines involved in their differentiation, of chemokines, and of chemokine receptors involved in their migration to CNS decrease after alemtuzumab administration, confirming that the long-term effect of alemtuzumab could rely on the reduction of both pro-inflammatory cytokines and T cell-recruitment into the CNS (7). However, mRNA levels of IL-1β, IL-6, TNF-α, IL-23, and IL-12 involved in Th17 or Th1 differentiation were higher compared to HS also after alemtuzumab administration. This phenomenon could relay in the peculiar ability of alemtuzumab to target specific immune cells. These cytokines are mainly produced by mature DCs that, in RMS, skew the immune response toward an autoreactive Th17 and Th1 phenotype (40). Thus, even though alemtuzumab treatment strongly depletes the pro-inflammatory T cells, this does not occur for DCs because of their low CD52 expression. A study by Gross and colleagues found a reduced number of circulating plasmacytoid-DC, a particular subset of DC able to elicit a pro-inflammatory immune response, at month 6 of alemtuzumab treatment, when compared to baseline, although the production of GM-CSF and IL-23 in these cells remained unchanged (41). In line with these observations, we could suggest an inflammatory persistence in these patients despite alemtuzumab. However, the mRNA amount of anti-inflammatory cytokines IL-10, TGF-β, and IL-27 is strongly upregulated and the ratio of pro/anti-inflammatory cytokines (not shown) significantly decreases after alemtuzumab and through to the end of the follow-up, indicating that, on the whole, alemtuzumab acts by shifting the immune response toward the production of anti-inflammatory cytokines. IL-27 production by DCs is an important inhibitor of Th17 and Th1 response (42). The production of the potent anti-inflammatory cytokine IL-10 by DCs is crucial for Treg induction. In the steady state, some of the peripheral Treg cells appear from peripheral CD4^+^CD25^−^FOXP3^−^ T cells that are exposed to antigen in the presence of TGF-β as well as IL-10 without IL-6 or IL-1β, which promote the upregulation of FoxP3 (43). In the same way, the mRNA level of FoxP3, the master regulator of the regulatory pathway in the development and function of Treg cells, was upregulated after alemtuzumab, reaching its highest values at month 24 and maintaining similar value ranges through to month 72.

Of particular meaning and interest are the results concerning the Treg cells. Several studies have shown a preferential expansion of the CD4+CD25^high^CD127^low^FoxP3+ Treg cells among the CD4+ cells at the early stage of recovery, reaching peak expansion at month 1 (6) and month 5 (38) and becoming significantly higher, compared to baseline, at month 24 (7). Our findings can now extend these observations to month 72, showing that the Treg cell percentage reverts back to its basal level, similar to the one observed in HS. They are mainly memory Treg cells that can specifically suppress the myelin-induced immune response starting from month 24, as in HS. These data show that the Treg cells increase in the CD4+ population occurs through homeostatic proliferation from the pool of lymphocytes that escape depletion rather than from new cells originating from non-depleted stem cells (6, 44, 45). Similarly, through classical Treg proliferation inhibition assay, other data showed a significant rebound proliferation at months 5 and 12 in PHA-activated PBMC depleted from the CD25 component (38) and the recovery of Treg cell competence at months 36 and 48 (44). Further evidence that supports the restoration of Treg cell suppressive ability was discussed by Gilmore and colleagues showing that the majority of Treg cells express CD39, an ectoenzyme able to promote and stabilize the functional capacity of Treg cells (38). Functional Treg cells can suppress pathogenic Th1 and Th17 responses, especially in the presence of high levels of IL-10 (46), which is also produced by Treg cells themselves. However, the reason why Treg cells recover their function to suppress autoreactive immune response during the repopulation period, as also occurs in HS, still needs to be investigated further, as it is unclear whether it is the result of an enhanced cytokine production on the part of the Treg cells themselves, rather than an altered composition and reactivity of repopulated CD4+ T cells that are more susceptible to regulation, or whether it is a combination of both.

Despite the fact that the primary objective of our study was to determine how CD4+ T cell subsets reconstitute after the administration of alemtuzumab, interestingly, data suggest that, although still early, there may be a role for Th17 and Treg cells in predicting the responsiveness of PwRMS to the treatment in question. The identification of immunological markers able to distinguish responding patients to the classical two courses of alemtuzumab from patients who will develop relapses, and therefore require further alemtuzumab infusions is one of the major challenges for neurologists when setting up the best therapy for their patients. The majority of papers addressing this concern have focused their studies on the identification of markers able to predict the appearance of clinical or radiological relapse (7, 38, 39). In all these reports, Th17 cells were shown to increase some months before the manifestation of the relapse, suggesting they are potential biomarkers. On the other hand, the percentage of Th17 cells in the PBMCs of our study is already higher at baseline in “non-responder” patients and is accompanied by a lower percentage of Treg cells, paving the way for further studies aimed at thoroughly immune profiling patients before starting the therapy. One of the major limitations of this study is indeed the relatively small number of subjects included. Nonetheless, our results represent a new piece of the puzzle concerning immunological reconstitution after alemtuzumab, and, piecing it all together with other similar ones, will be the basis for the correct design of further studies.

Summarizing therefore, our data confirm that the efficacy of alemtuzumab is associated with a reshuffle of the CD4+ immune response from pro- to anti-inflammatory, more or less in line with that of a healthy subject. Besides quantitative changes of the cell repertoire, qualitative alterations of CD4+ T cell subsets were also observed and can be described through two major phenomena: on the one hand, there is a durable decrease of the inflammatory pathways characterized by the disappearance of Th17 and Th1 self-reactive responses, the reduced expression of master regulator factors, and the cytokines related to those subsets and of chemokines and their receptor-connected to CNS recruitment. On the other hand, the restoration of Treg cell suppressor function and the increase of anti-inflammatory cytokines contribute to immune-tolerance promotion versus CNS antigens. Overall, this peculiar mode of action of alemtuzumab is reflected in its durable effect that is maintained for up to 6 years, without the need for further treatment during that period.

## Data Availability Statement

The original contributions presented in the study are included in the article/**Supplementary Material**. Further inquiries can be directed to the corresponding author.

## Ethics Statement

The studies involving human participants were reviewed and approved by Comitato Etico Interaziendale, AOU San Luigi Gonzaga. The patients/participants provided their written informed consent to participate in this study.

## Author Contributions

SR and SD: designed and conceptualized the study; interpreted the data; drafted the manuscript for intellectual content. VB: major role in the acquisition of data and analyzed the data. AM, DT, and FN: interpreted the data; revised the manuscript for intellectual content. EC, AV, MH, IA, PA, and DH: patient enrolment and follow-up; revised the manuscript for intellectual content. MC: designed and conceptualized the study; revised the manuscript for intellectual content. All authors contributed to the article and approved the submitted version.

## Funding

This study was partially supported by Genzyme (Bio2009001) and the Federazione Italiana Sclerosi Multipla (FISM, 2011/R/28). None of the funding sources had a role in the study design; collection, analysis, and interpretation of data; or the decision to submit the paper for publication.

## Conflict of Interest

The authors declare that the research was conducted in the absence of any commercial or financial relationships that could be construed as a potential conflict of interest.

## Publisher’s Note

All claims expressed in this article are solely those of the authors and do not necessarily represent those of their affiliated organizations, or those of the publisher, the editors and the reviewers. Any product that may be evaluated in this article, or claim that may be made by its manufacturer, is not guaranteed or endorsed by the publisher.
