# Supplementary material for: Long-Term Effects of Alemtuzumab on CD4+ Lymphocytes in Multiple Sclerosis Patients: A 72-Month Follow-Up
Source: Front Immunol. 2022 Feb 28;13:818325. doi: 10.3389/fimmu.2022.818325 (PMC8919044; doi:10.3389/fimmu.2022.818325)
Supplement: Supplementary file 1 [file DataSheet_1.pdf]

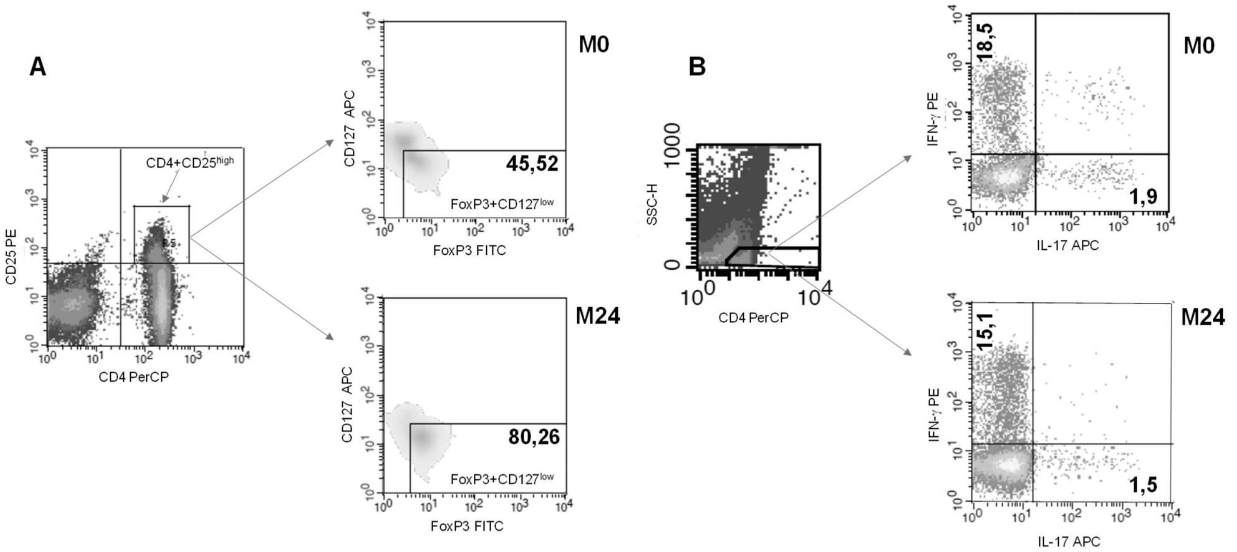

Supplementary Figure 1. Gating strategies for Treg cells **(A)** and Th17 and Th1 cells **(B)**. Representative dots plots were shown for PwRMS before treatment (M0) and after 24 months (M24).

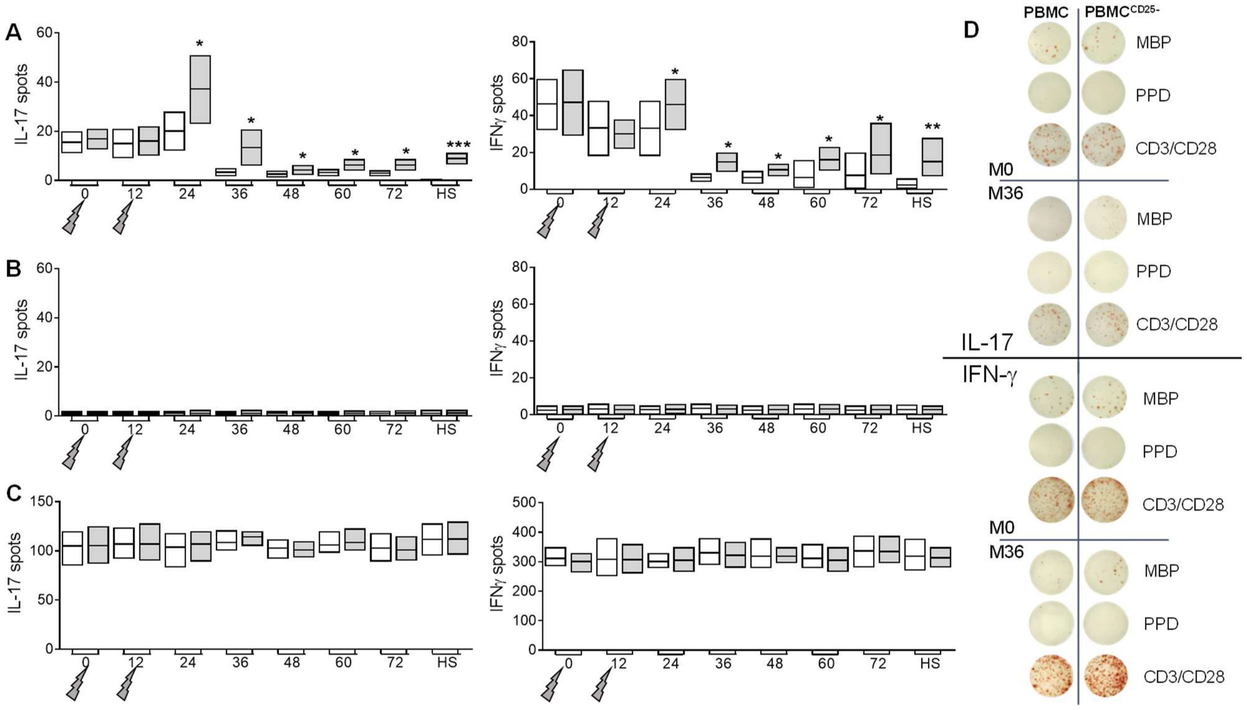

Supplementary Figure 2. MBP **(A)**, PPD **(B)** and anti-CD3/CD28 **(C)** specific spots in the PBMC (white bars) or in the $PBMC^{CD25-}$ (grey bars) after background subtraction of unstimulated PBMC or $PBMC^{CD25-}$. Bars represent mean, minimum and maximum values. *$p<0.05$, **$p<0.001$, ***$p<0.0001$, Pearson t test. Months after alemtuzumab administration and HS are indicated in the X axis. The arrows indicate infusion of alemtuzumab. **(D)** Representative IL-17 and IFN-γ spots in PBMC (on the left) and in $PBMC^{CD25-}$ (on the right) at month 0 and 36.
